# Increased stem cells delivered using a silk gel/scaffold complex for enhanced bone regeneration

**DOI:** 10.1038/s41598-017-02053-z

**Published:** 2017-05-19

**Authors:** Xun Ding, Guangzheng Yang, Wenjie Zhang, Guanglong Li, Shuxian Lin, David L. Kaplan, Xinquan Jiang

**Affiliations:** 1 Department of Prosthodontics, Ninth People’s Hospital affiliated to Shanghai Jiao Tong University, School of Medicine, 639 Zhizaoju Road, Shanghai, 200011 China; 2 Oral Bioengineering and regenerative medicine Lab, Shanghai Research Institute of Stomatology, Ninth People’s Hospital Affiliated to Shanghai Jiao Tong University, School of Medicine, Shanghai Key Laboratory of Stomatology, 639 Zhizaoju Road, Shanghai, 200011 China; 3 Department of Oral and Maxillofacial Surgery, Ninth People’s Hospital affiliated to Shanghai Jiao Tong University, School of Medicine, 639 Zhizaoju Road, Shanghai, 200011 China; 4 Department of Biomedical Engineering, School of Engineering, Tufts University, 4 Colby St, Medford, MA 02155 USA

## Abstract

The low *in vivo* survival rate of scaffold-seeded cells is still a challenge in stem cell-based bone regeneration. This study seeks to use a silk hydrogel to deliver more stem cells into a bone defect area and prolong the viability of these cells after implantation. Rat bone marrow stem cells were mingled with silk hydrogels at the concentrations of 1.0 × 10^5^/mL, 1.0 × 10^6^/mL and 1.0 × 10^7^/mL before gelation, added dropwise to a silk scaffold and applied to a rat calvarial defect. A cell tracing experiment was included to observe the preservation of cell viability and function. The results show that the hydrogel with 1.0 × 10^7^/mL stem cells exhibited the best osteogenic effect both *in vitro* and *in vivo*. The cell-tracing experiment shows that cells in the 1.0 × 10^7^ group still survive and actively participate in new bone formation 8 weeks after implantation. The strategy of pre-mingling stem cells with the hydrogel had the effect of delivering more stem cells for bone engineering while preserving the viability and functions of these cells *in vivo*.

## Introduction

Stem-cell-based bone regeneration was recently considered to be a preferable method to reconstruct bone defects compared to autologous bone grafts or allogeneic bone grafts, which have obvious flaws such as requiring extra surgery or potentially producing a severe immune response^1–4^. Seeded stem cells initiate local bone formation with biomaterials as carriers^5–7^. The *in vivo* osteogenic differentiation potential of seeded bone mesenchymal stem cells (BMSCs) has been indicated by various studies^8–10^. However, along with the effort to enhance the osteoconductivity of the biomaterials, some researchers have shown that the seeded stem cells exhibit a low survival rate after implantation *in vivo* ^11–15^. Meanwhile, the uncertainty of the *in vivo* cell fate and the failure of some clinical trials caused by cell deficiency remain unsolved questions regarding clinical approaches^16–18^. Strategies to improve this situation rely on exploiting innovative biomaterials that could amplify the initial seeding quantity of stem cells while possessing excellent cytocompatibility for maintaining the seeded cells’ viability.

Silk fibroin is a biodegradable material with strong mechanical properties, superior biocompatibility, a simple fabrication process and tunable processing parameters^19–22^; above all, it can be easily fabricated into various forms, such as sponges, films, fibers or gels^23, 24^. Our previous study successfully fabricated this material into a hydrogel and scaffold; both have exhibited excellent biocompatibility because they have successfully improved bone regeneration by delivering stem cells and growth factors to the defect area^25, 26^. Notably, the silk-fibroin-based hydrogel allows stem cells to be encapsulated before gelation, and, as a highly hydrated material, it can suspend cells homogeneously within the hydrogel network. This distinguished capacity of the silk hydrogel renders it as a possible carrier to deliver a large quantity of cells to the bone regeneration area^27–30^.

**Figure 1:**
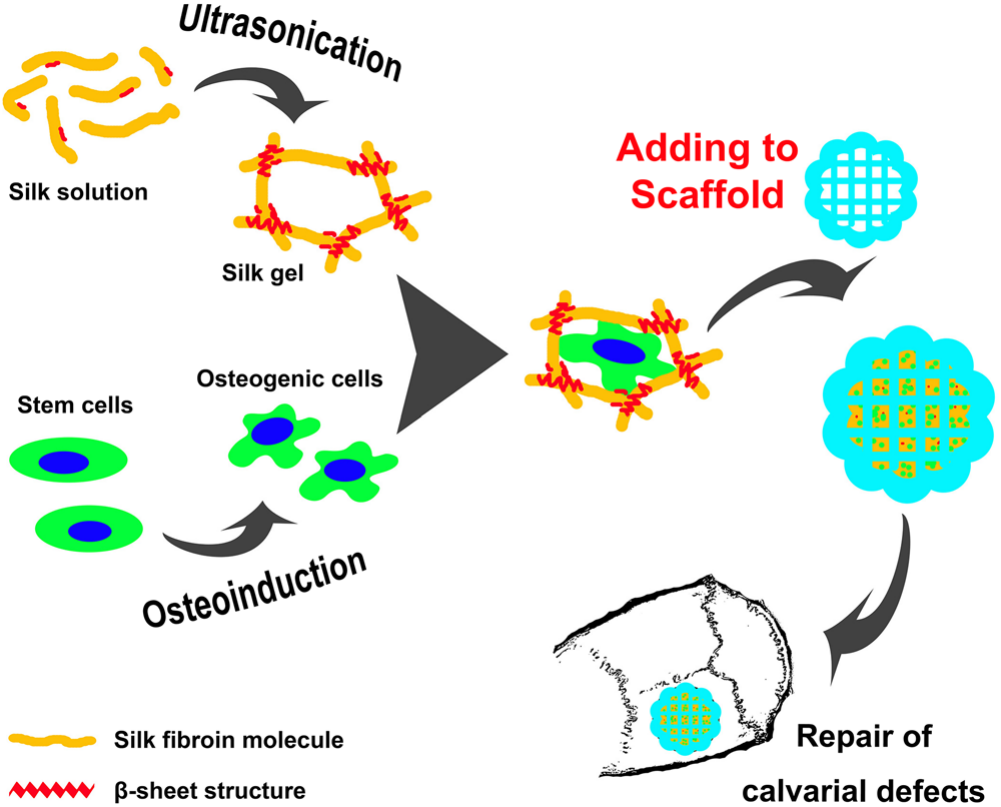
Schematic illustration. Schematic illustration of the fabrication protocol of the cell containing silk gel/scaffold complex. Silk solution was ultrasonicated to initiate the formation of β-sheet structure. Meanwhile, the solution was mixed with osteogenic cells, and the well-mixed solution was added dropwise to a silk scaffold. After gelation, the cell-carrying silk scaffold was ready for implantation to repair rat calvarial defects.

In the present study, a silk hydrogel was used to deliver more bone marrow stem cells (BMSCs) into the porous three-dimensional (3D) scaffolds to enhance bone regeneration, where the mass of stem cells carried by the silk hydrogel could grow inside the specifically shaped scaffold. *In vitro*, we tested the transportation rates of small molecules and large proteins into the gel, which might enhance the survival rate of inner seeded BMSCs. After osteogenic induction *in vitro*, the silk gel/scaffold complex containing 1.0 × 10^7^/mL stem cells was implanted into critical-sized calvarial defects in rats to evaluate the bone regeneration effects. The schematic illustration of this strategy is shown in Fig. 1.

## Results

### Nutrient transportation performance of the silk gel/scaffold complex

**Figure 2:**
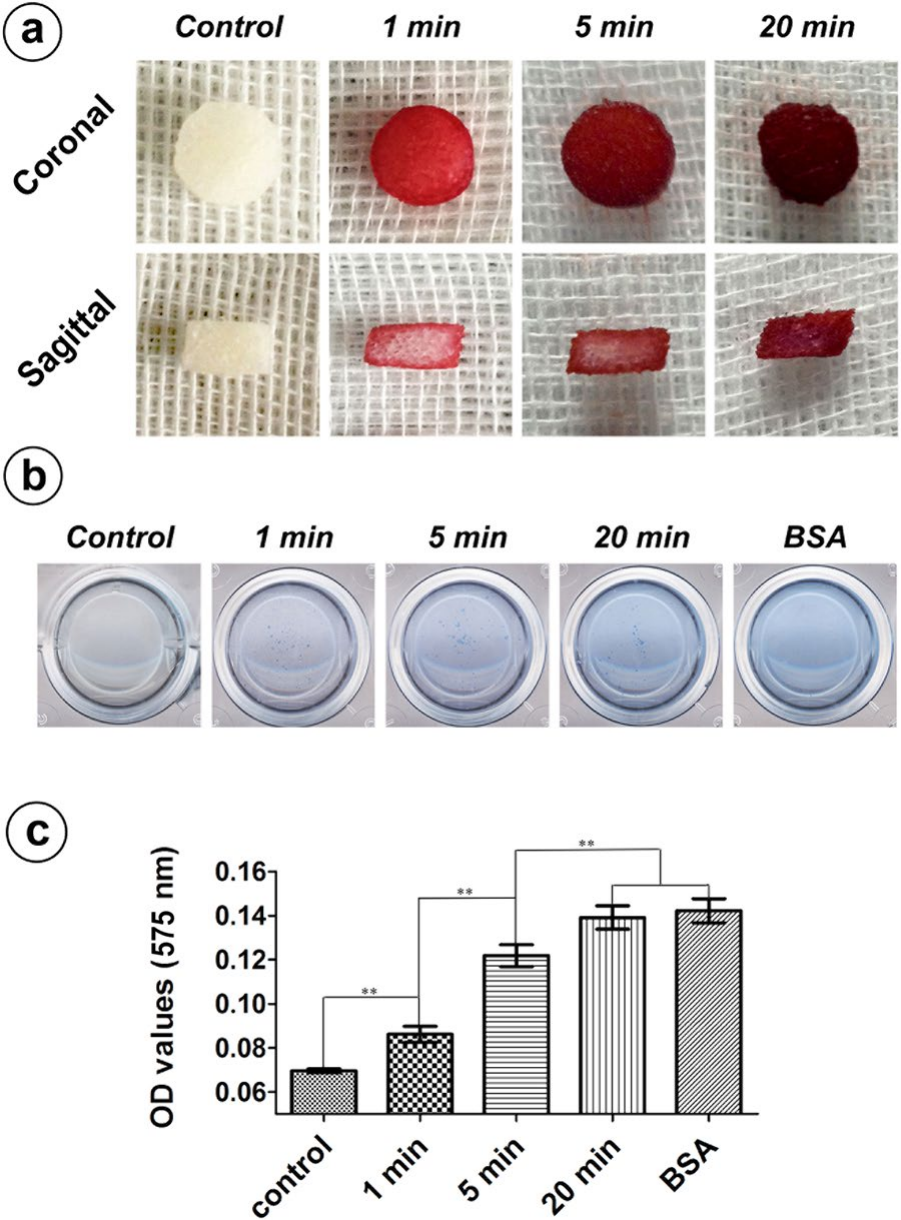
Nutrient transportation. Nutrient transport through the silk gel/scaffold complex. (**a**) Silk gel/scaffold complex was immersed in alizarin red solution and removed at different time points. The gel/scaffold complex was cut from both the coronal and sagittal angles to see the permeation condition of the alizarin red solution. (**b**) and (**c**) Silk gel/scaffold complex were immersed in BSA solutions for different periods (1 min, 5 min and 20 min) and removed to release BSA in distilled water for 24 h. The distilled water that contained released BSA were further reacted with BSA working solution, and their OD value were measured. (**Represents *p* < 0.01).

This experiment was carried out after the cell-free silk hydrogel was added dropwise to the scaffold (the SEM images and FT-IR spectra of the silk scaffold can be found online as Supplementary Fig. S1 and Supplementary Fig. S2, respectively). The results showed that the alizarin red solution can consistently permeate through the silk gel/scaffold complex. At 1 min and 5 min, the alizarin red solution permeates gradually from every interface to the core of the complex. From gross observation, the alizarin red solution almost went through the complex in 20 min from both the sagittal and coronal angles (Fig. 2a). The silk gel/scaffold complex could also absorb and release large protein molecules, such as bovine serum albumin (BSA), in a short period of time. The OD value of the 1 min group was significantly higher that of the control group (*p* < 0.01), which indicates that the BSA was rapidly absorbed by the complex from the first minute. In addition, there were also significant differences between the 1 and 5 min groups and the 5 and 20 min groups *(p* < 0.01). These results indicate a consistent absorbance of BSA by the silk gel/scaffold complex during the first 20 min. Noticeably, there is no significant difference between the 20 min group and the pure BSA group, where we speculate that the complex could almost thoroughly absorb the BSA in 20 min (Fig. 2b and c).

### Cell interactions within the hydrogel

**Figure 3:**
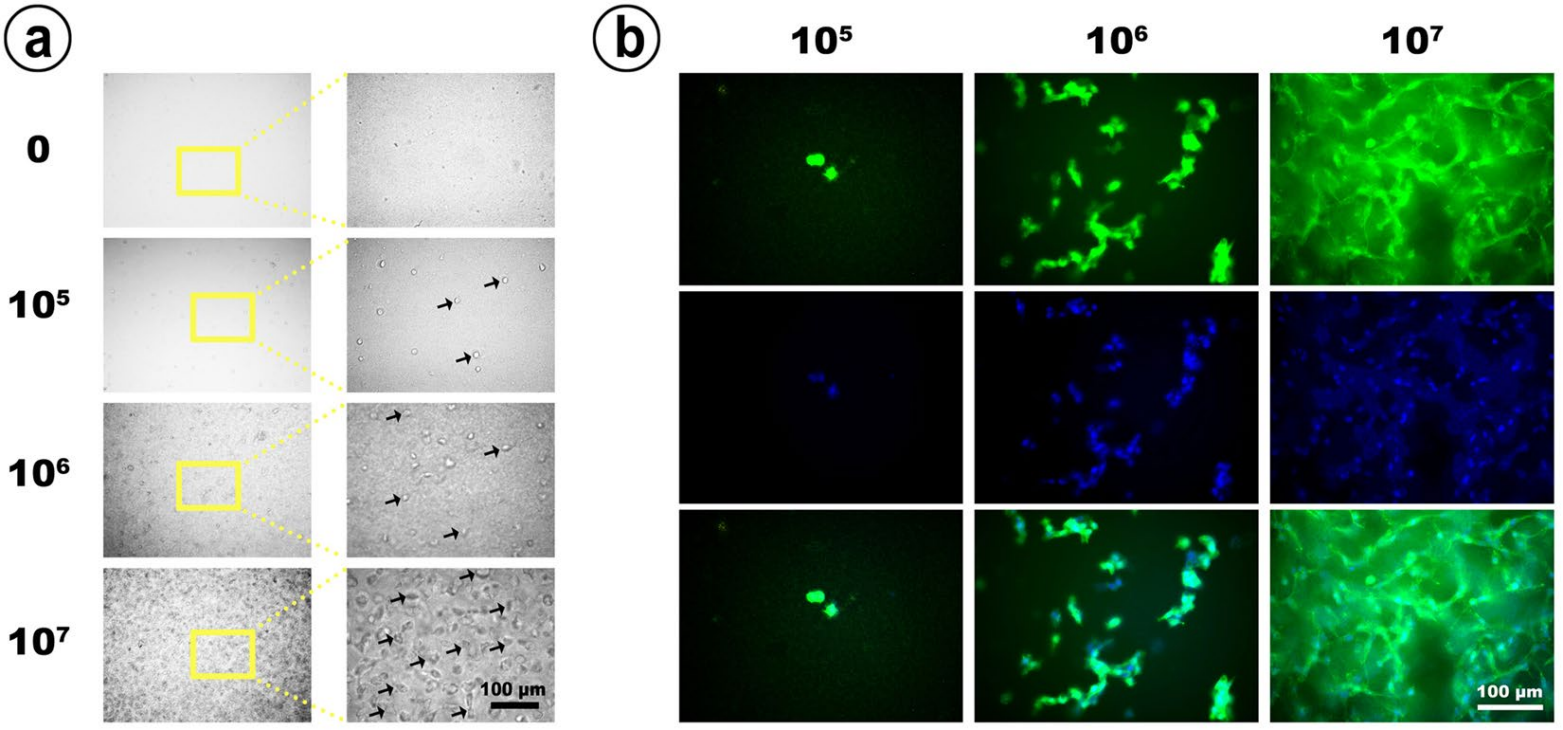
Cell in gel. (**a**) Silk hydrogels contained different densities of cells and were observed under a microscope. The yellow frames show typical areas, and the black arrows indicate encapsulated cells. (**b**) Different densities of hydrogel-enwrapped cells co-stained with FITC-phalloidin and DAPI and observed under a confocal laser scanning microscope.

Both microscopy and confocal laser scanning microscopy (CLSM) showed the intensiveness of the cell distribution in the 1.0 × 10^7^ group compared to the 1.0 × 10^5^ group or the 1.0 × 10^6^ group (Fig. 3). More importantly, CLSM indicated that both the 1.0 × 10^5^ and 1.0 × 10^6^ groups had almost no cell interactions, that is, cells were independently dispersed within the hydrogel (Fig. 3a). However, for the 1.0 × 10^7^ group, CLSM showed a high rate of cell–cell interaction, where most cells were connected to other cells and present as a network (Fig. 3b). In the 1.0 × 10^7^ group, the entire silk gel was well distributed, with cells that could produce local calcium deposition and mineralization even in the very core of the hydrogel.

### Cell proliferation

**Figure 4:**
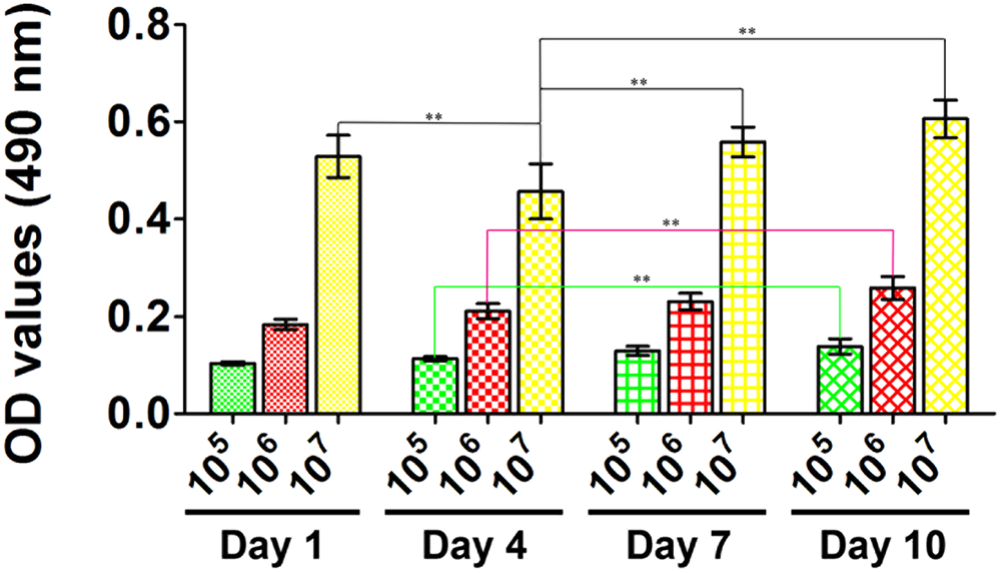
MTT. MTT assay of cells incubated in silk hydrogel for 1, 4, 7 and 10 days. (*Represents *p* < 0.05; **Represents *p* < 0.01).

Both the 1.0 × 10^5^ group and the 1.0 × 10^6^ group underwent a stable increase in cell quantity from day 1 to day 10. Significant differences were detected between day 4 and day 10 for these two groups (*p* < 0.01). For the 1.0 × 10^7^ group, cell quantity declined after initial seeding, with significant differences detected between day 1 and day 4 (*p* < 0.01). However, the cell quantity then consistently increased until day 10. Statistically significant differences that indicated this increase were also detected between day 4 and day 7 as well as day 4 and day 10 (*p* < 0.01). Finally, the cell quantities in the 1.0 × 10^7^ group showed no significant differences between day 1 and day 10 (Fig. 4).

### Osteogenic potential *in vitro*

**Figure 5:**
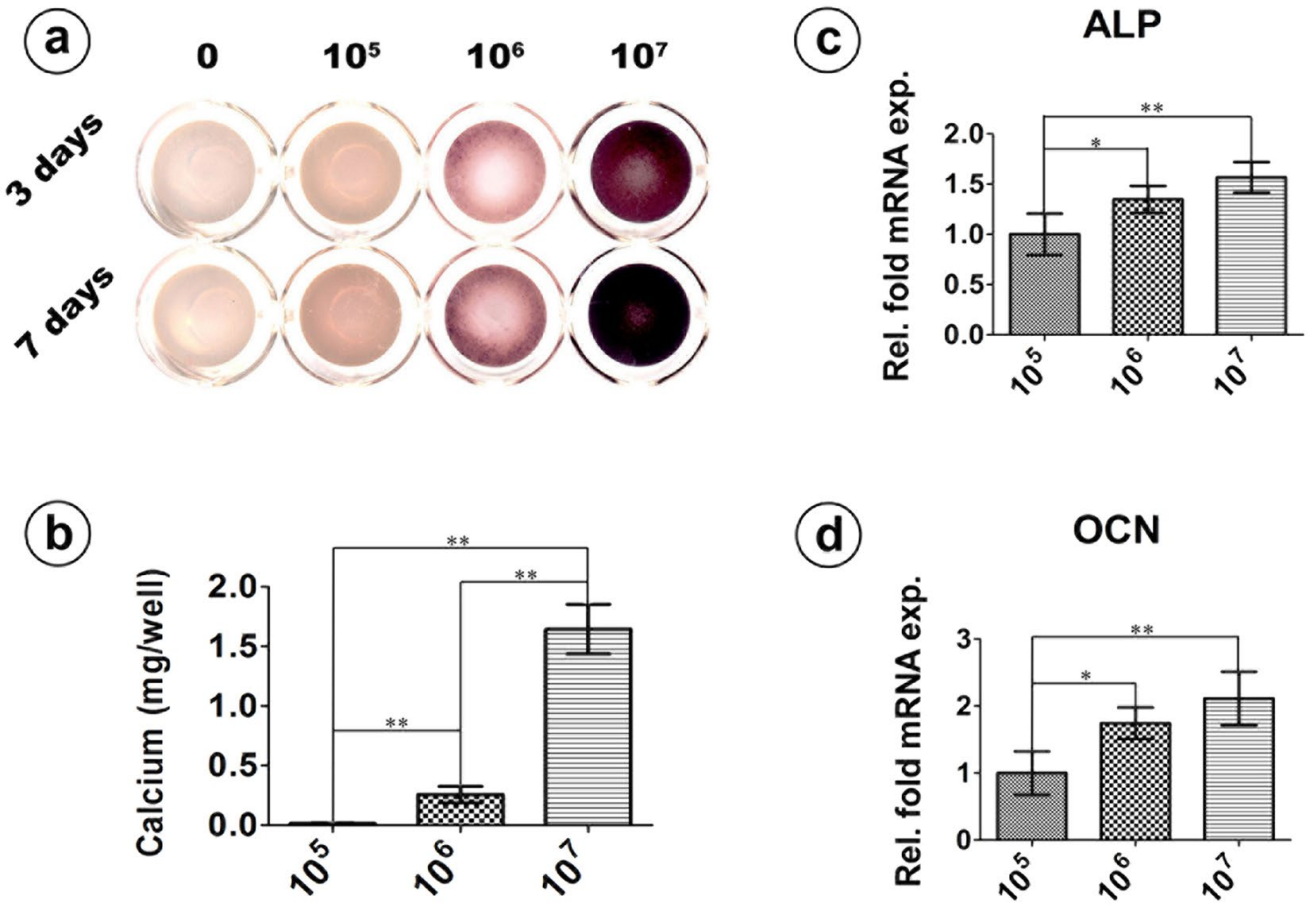
ALP-qPCR. *In vitro* osteogenic potential evaluation of different densities of encapsulated cells. (**a**) ALP staining of different densities of cell-containing silk hydrogels incubated *in vitro* for 3 and 7 days. (**b**) Calcium deposition assay at day 21. (**c**) and (**d**) Osteogenic gene expression of different groups of cells at day 7. (*Represents *p* < 0.05; **Represents *p* < 0.01).

Both the alkaline phosphatase (ALP) activity assay and calcium deposition assay showed significant differences among the three groups. In the ALP activity assay, the 1.0 × 10^7^ group appeared to have the highest ALP expression, followed by the 1.0 × 10^6^ group and the 1.0 × 10^5^ group (Fig. 5a). The calcium deposition in the 1.0 × 10^7^ group was 1.6458 ± 1.1770 mg/well, which was significantly higher than that in the 1.0 × 10^6^ group (0.2575 ± 0.028 mg/well) and the 1.0 × 10^5^ group (0.0143 ± 0.002 mg/well) (*p* < 0.01). There was also a statistically significant difference between the 1.0 × 10^5^ group and the 1.0 × 10^6^ group (*p* < 0.01) (Fig. 5b). The results of the real-time quantitative polymerase chain-reaction (qPCR) assay are presented relative to the value for the 1.0 × 10^5^ group. There were statistically significant differences in the expression of both ALP and osteocalcin (OCN) genes between the 1.0 × 10^5^ group and the 1.0 × 10^6^ group (*p* < 0.05), and more significant differences between the 1.0 × 10^5^ group and the 1.0 × 10^7^ group (*p* < 0.01) (Fig. 5c and d).

### Micro-CT

**Figure 6:**
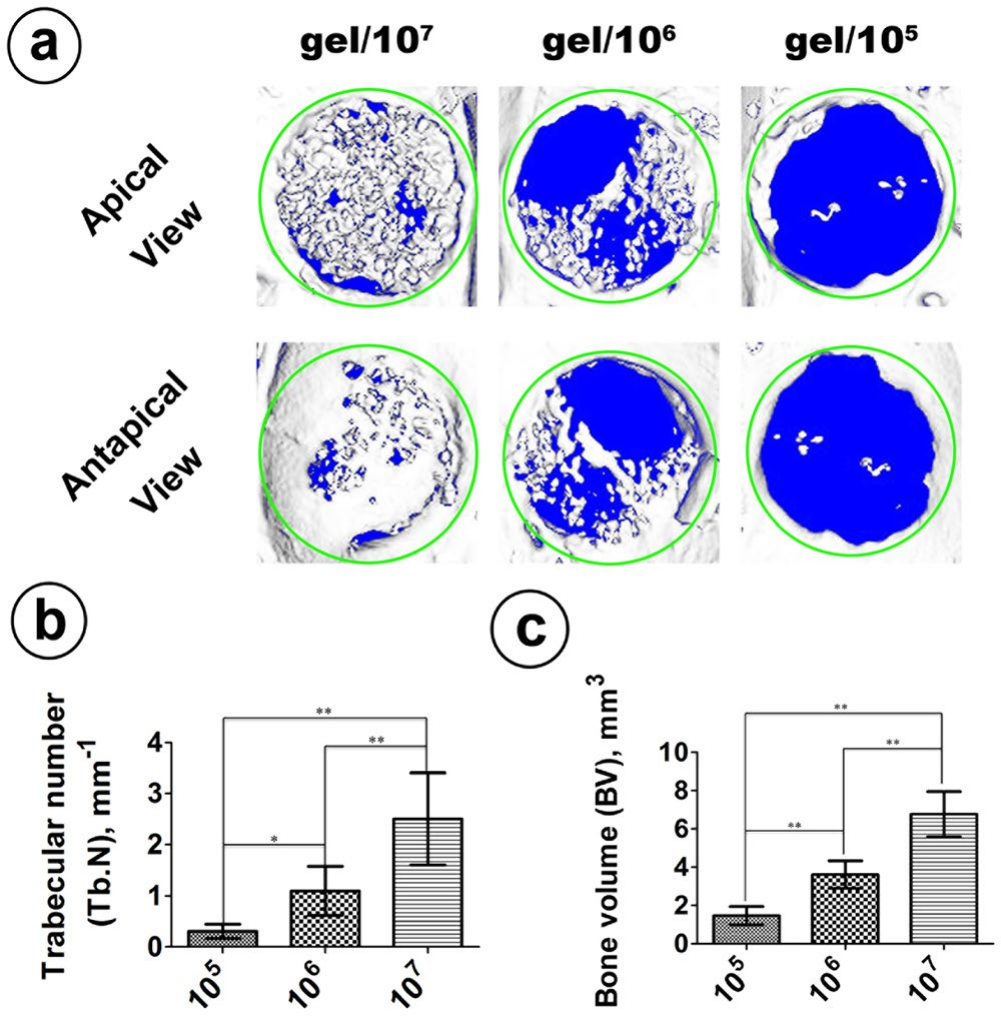
Micro-CT. Micro-CT images of the rat calvarial defect area after 8 weeks of implantation. (**a**) Representative image of the newly formed bone at the defect area from both apical and antapical views. Green circles indicate the calvarial defect area. (**b**) Quantitative morphometric analysis of the newly formed trabecular number. (**c**) Quantitative morphometric analysis of the new bone volume. (*Represents *p* < 0.05; **Represents *p* < 0.01).

Figure 6a shows the reconstructed image of the newly formed bone in the rat calvarial defect area obtained using Micro-CT from both the apical and antapical views. In contrast to the 1.0 × 10^5^ group and the 1.0 × 10^6^ group, which exhibited few calcium nodules with a large amount of vacancy in the defect area, the 1.0 × 10^7^ group showed significantly higher new bone volume (6.767 ± 0.481 mm^3^) and the highest trabecular numbers (1.098 ± 0.197) (*p* < 0.01) after implantation *in vivo* for 8 weeks, where the newly formed calcium nodules grew evenly all over the defect area and connected to each other as a network (Fig. 6a, b and c). There were also statistically significant differences between the 1.0 × 10^5^ group and the 1.0 × 10^6^ group with respect to both new bone volume (*p* < 0.01) and trabecular number (*p* < 0.05).

### Histological analysis

**Figure 7:**
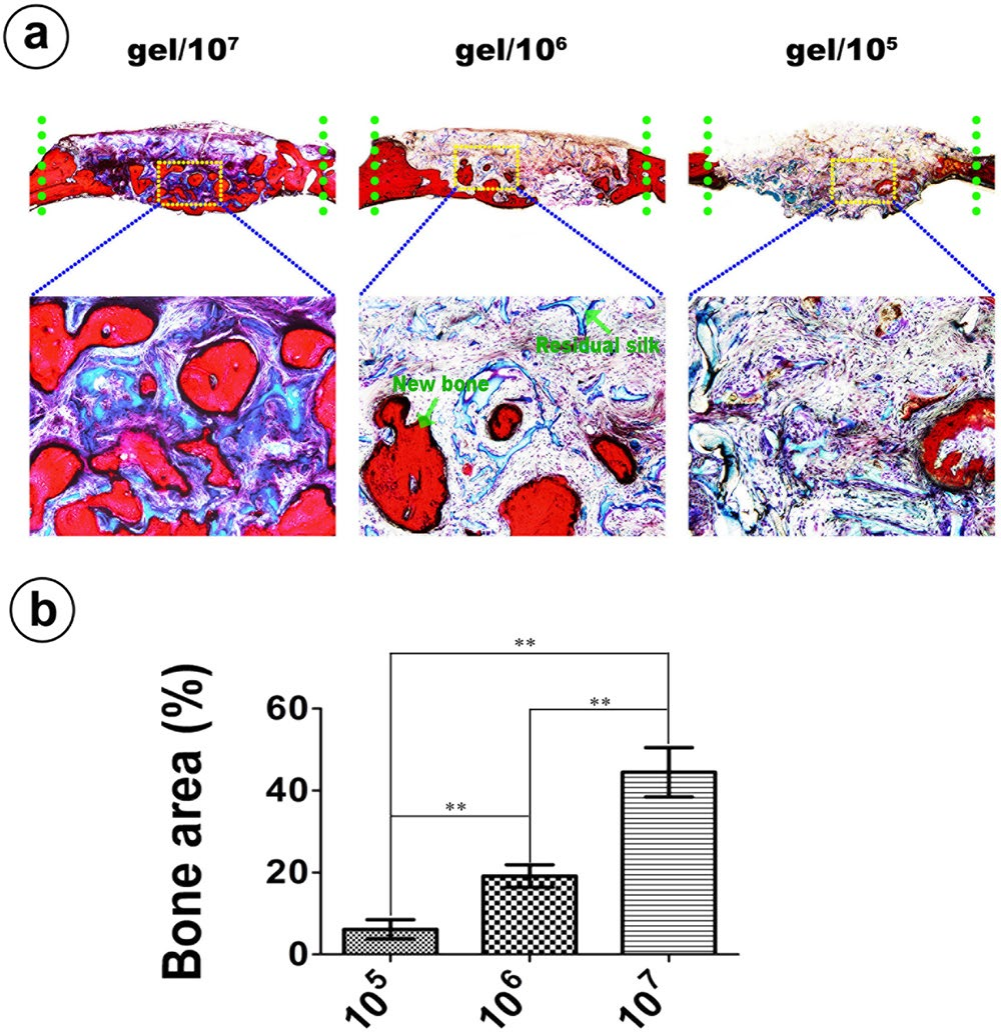
VG Staining. VG-stained histological sections. (**a**) The green dot line shows the calvarial defect area. The yellow frames show the representative area of the new bone formed by cell carrying silk gel. (**b**) Quantitative morphometric analysis of the new bone area. (**Represents *p* < 0.01).

Figure 7 shows the histological sections stained with Van Gieson’s picro fuchsin staining. Consistent with the results of Micro-CT, the 1.0 × 10^7^ group demonstrated the highest amount of new bone area (44.45 ± 2.461%), notably higher than the 1.0 × 10^6^ group (19.133 ± 1.112%) (*p* < 0.01). Mostly occupied by the remnant silk hydrogel, the 1.0 × 10^5^ group presented a percentage of new bone area of approximately 6.236 ± 1.172%, which was significantly lower than that for the 1.0 × 10^6^ group and the 1.0 × 10^7^ group (*p* < 0.01).

### Cell tracing

**Figure 8:**
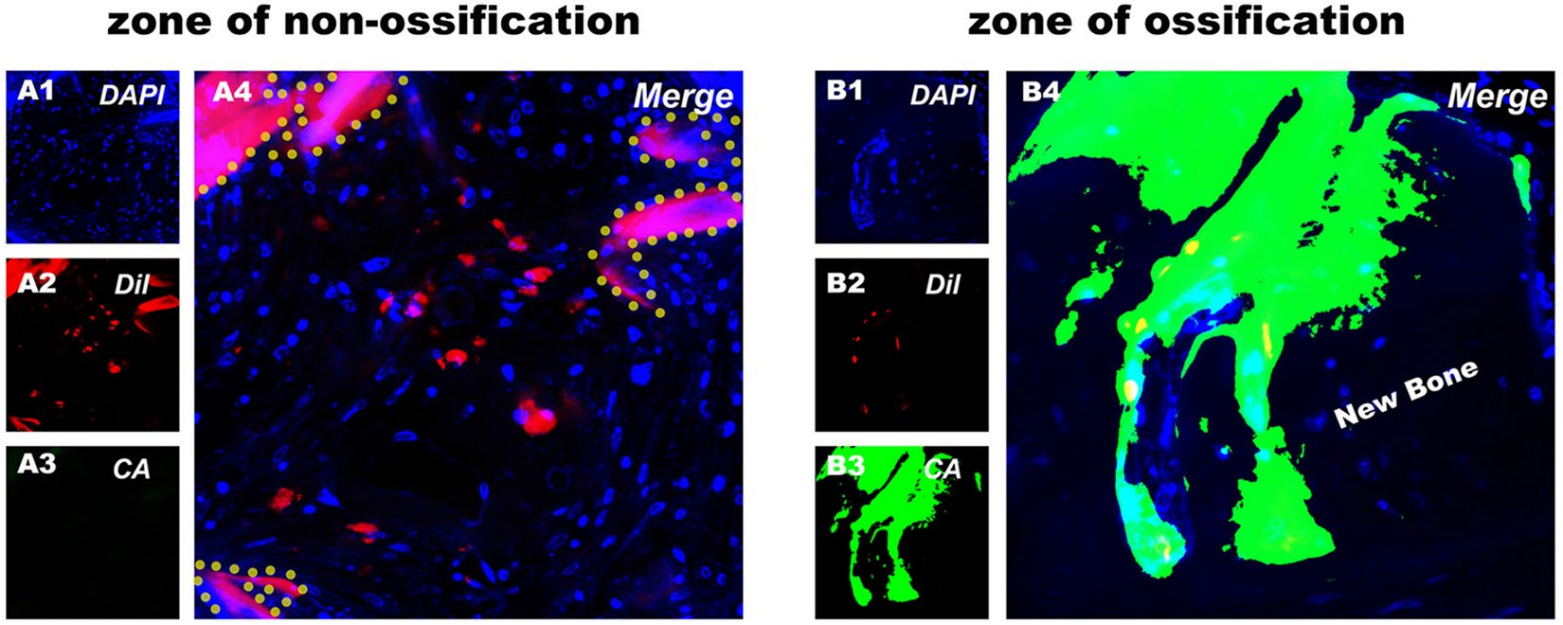
Cell tracing. Representative histological analysis of CM-Dil labeled BMSC carrying silk gel/scaffold complex implanted in a rat calvarial defect for 8 weeks. (**A1**) and (**B1**) DAPI staining. (**A2**) and (**B2**) CM-Dil labeled BMSCs. (**A3**) and (**B3**) Bone formed between 6 weeks and 8 weeks labeled by calcein.

The CM-Dil labeled BMSCs were still surviving after 8 weeks of implantation in both ossification and non-ossification zones. In the ossification zone, the calcein labeling area (green) indicated the new bone that formed between 6 weeks and 8 weeks after implantation, where the labeled cells were actively participating in this procedure (Fig. 8).

## Discussion

Stem-cell-based bone engineering is very promising and opens the possibility of developing cell-carrying materials with optimized abilities to carry as many cells as possible and prolonged lifespans for exerting certain functions *in vivo*. The conventional cell seeding process relies on the cell to adhere to the scaffold. In addition to the initial seeded cell quantity used in another study, which varied from 1.0 × 10^5^ to 5.0 × 10^5^, the seeding efficiency was only 50% to 70% because most of the stem cells adhered only at the scaffold’s surfaces and easily fell off^31–34^. Therefore, the actual seeding quantity *in vivo* is beyond prediction. We see this utterly inadequate and unstable cell seeding quantity as a huge challenge, combined with the more difficult problem of maintaining the cell quantity for a long period of time after implantation.

Based on the evidence that silk fibroin possesses excellent mechanical properties, fine biocompatibility and a controllable degradation rate, scientists have been incubating various cell lines with silk fibroin in the form of films, fibers or porous scaffolds for bone or soft tissue engineering^35–39^. Among all of those forms made using silk fibroin, we found hydrogel to be an ideal cell carrier because of its high water content, adequate mechanical strength and easily controlled gelation process^27–30^. More importantly, the manufacturing process for producing silk hydrogel allows the mingling of a determined quantity of cells before gelation, which could ensure the initial cell seeding number and allow for optimization. In this study, we quantitatively seeded selective densities of rat BMSCs into the silk hydrogels. The maintained cell viability in the three experiment groups (Fig. 4) demonstrated the marked cytocompatibility of the silk hydrogel.

We compared the osteogenic potential of these cell containing silk hydrogels and found that silk gels encapsulated with higher cell quantities tended to have better osteogenic potential both *in vitro* and *in vivo*. The 1.0 × 10^7^ group exhibited the highest ALP activity, the most calcium deposition and the strongest osteogenic-related gene expression *in vitro*. Consistent with the *in vitro* experiment, the 1.0 × 10^7^ group showed a clear increase in bone formation *in vivo* (Figs 6 and 7). Micro-CT showed that the 1.0 × 10^7^ group had more and faster calcium deposition from the seeded BMSCs, which led to a prominent performance with respect to both new bone volume and trabecular number in the defect area compared to the other two groups (Fig. 6). The histological analysis also confirmed this result, as Van Gieson’s staining for the 1.0 × 10^7^ group showed the highest ratio of new bone area (Fig. 7). To further confirm that the rapid bone regeneration was produced by the encapsulated BMSCs, we conducted a cell tracing experiment that showed that the encapsulated stem cells maintained their viability and actively participated in the local bone formation process after implantation (Fig. 8).

This comparison of the three experimental groups verifies that the excellent bone formation was primarily a result of the larger quantity of encapsulated stem cells. Generally, the 1.0 × 10^7^ group exhibited more mineralization locations in the defect area, providing more cores for calcium nodule formation. More importantly, the long-term *in vivo* preservation of the viability and function of the encapsulated stem cells in the 1.0 × 10^7^ group guaranteed a continuous bone formation process. It is interesting that the expression of osteogenesis-related genes (ALP and OCN) in the 1.0 × 10^7^ group was detected to be the strongest among the three groups (Fig. 5), which implies the better osteogenic differentiation potential of single cells when they are in a higher degree of encapsulation. We speculated that the result may be attributed to the enhanced cell–cell interactions in the 1.0 × 10^7^ group (Fig. 3), where this optimization could increase the formation of gap junctions that directly transfer signaling molecules and metabolites between adjacent cells^40–42^. In addition, the increased cell quantity may also lead to more cytokines and proteins being secreted into the microenvironment that stimulate cell behavior^43, 44^.

Conventional cell scaffolds rely on the seeded cells to grow into the center of the defect area, in which the long-term repair process and the insufficient oxygen and nutrients inside the scaffolds make it a challenge to preserve the survival of implanted cells^45–48^. However, the strategy of mingling stem cells with a silk hydrogel before gelation ensures that the cells are homogenously suspended in the material, which leads to mineralization of the entire defect area and reduction of the repair time. In addition, the silk hydrogel has a permeability that allows the transfer of both small molecules and proteins (Fig. 2) that could nourish the seeded cells to preserve their viability after implantation. This study emphasized the crucial role of stem cells in rapid bone regeneration while confirming the cell carrying properties and *in vivo* long-term cell viability supporting properties of the silk gel/scaffold complex.

The fast and efficient bone-forming property achieved by the stem-cell-carrying silk hydrogel could be applied to multiple shapes of small defects by simply changing the nature or material of the scaffolds. We have now applied this strategy only to small defect areas to confirm the encapsulated cell functions with respect to rapid bone regeneration; verification of its applicability to large defect repair is still needed. Notably, achievements in *in vitro* osteogenic induction of hydrogel-enwrapped stem cells also hint at the possibility of pre-inducing the encapsulated stem cells into osteogenic progenitor cells to attain calcium deposition and local mineralization before implantation, which might further shorten the time needed for bone regeneration and reduce the risk of *in vivo* cell necrosis.

## Methods

### Animals

The animals used in this study were all obtained from the Ninth People’s Hospital Animal Center (Shanghai, China) for both the calvarial defect repair experiment and BMSC isolation and culture. All animal experiments were conducted in accordance with the regional Ethics Committee guidelines, with the protocols approved by the Animal Care and Experiment Committee of Ninth People’s Hospital.

### Rat BMSC isolation and culture

BMSCs were obtained and cultured from 4-week-old male F344 rats, as we previously published^26, 49^. Briefly, after euthanizing the rats with an overdose of pentobarbital injected intraperitoneally, the femurs were separated with the epiphysis being cut off. The marrow was then quickly rinsed out using Dulbecco’s modified Eagle’s medium (DMEM; Gibco, USA) containing 10% (v/v) fetal bovine serum (FBS; Gibco, USA). The isolated BMSCs were cultured in Dulbecco’s modified Eagle’s medium with 10% (v/v) fetal bovine serum. Cells were incubated at 37 °C in an environment containing 5% CO_2_. Non-adherent cells were removed by changing the medium after 24 h. When the confluence reached 80–90%, the BMSCs were subcultured at a density of 1.0 × 10^5^ cells/mL with trypsin-ethylenediamine tetra-acetic acid (EDTA, 0.25% w/v trypsin, 0.02% EDTA). Cells at passage 2–3 were collected and resuspended in DMEM for subsequent cell encapsulation.

### Preparation of the materials

Purified silk fibroin stock solutions were prepared at 8.0 wt% with deionized water diluted to approximately 4.0 wt% and used in the subsequent studies, as previously described^25, 26, 50, 51^. The sterilized silk fibroin solution sterile DMEM powder was blended and sonicated to initiate gelation; approximately 10 min was required for the solution to fully transform into a hydrogel. Before it turned into a gel, a certain volume of cell suspension was added into the silk solution to reach three different final concentrations of 1.0 × 10^5^ cells/mL, 1.0 × 10^6^ cells/mL and 1.0 × 10^7^ cells/mL. To observe the cell conditions inside the silk gel and evaluate their proliferation and osteogenic differentiation abilities *in vitro*, 20 μL of the mixed solutions was added dropwise to 96 well-plates and incubated at 37 °C for 10 min for gelation before conducting *in vitro* experiments. In addition, 20 μL of the silk gel was added dropwise to a porous silk scaffold (pore sizes 350–420 μm, 5 mm in diameter and 2 mm in thickness)^26^ to evaluate the transfusion condition of this silk gel/scaffold complex. For the *in vivo* rat calvarial repair experiment, different densities of cell-containing silk gels were added dropwise to the silk scaffold for gelation, and then they were incubated in osteogenesis-induced medium for 7 days before *in vivo* implantation.

### Nutrient transportation performance of the silk gel/scaffold complex

After the silk gels were fully gelled in the silk scaffolds, the gel/scaffold complexes were immersed in alizarin red solution and removed at selected time points (1 min, 5 min and 20 min) to observe the transfusion condition of the alizarin red solution from both the coronal and sagittal angles. To further evaluate the ability to transport large protein molecules, we placed the gel/scaffold complex in a-24 well plate containing 1 mL of distilled water and 20 μL of BSA (0.5 mg/mL) in each well, making the final BSA concentration of the immersed solution to be 10 μg/mL (the control group well contained only 20 μL of distilled water). After immersing the complex in the plate for different time periods (1 min, 5 min and 20 min), the gel/scaffold complex was removed and washed with PBS 3 times to remove the redundant BSA. Each gel/scaffold complex was then placed into 1 mL of distilled water at 4 °C overnight to release the encapsulated BSA (20 μL, 10 μg/mL BSA solution was added to 1 mL of distilled water to serve as positive control in this study). A mixture of bicinchoninic acid and copper sulfate solution was added into each well as the BCA working solution (Beyotime, Shanghai, China). The plate was then incubated at 37 °C for 30 min. The quantitative measurement of released BSA was present as the optical density (OD) value of the solutions at a length of 630 nm by an ELX ultra microplate reader (BioTek, Winooski, VT). Both transfusion experiments included only cell-free silk gel/scaffold complexes.

## Analysis of BMSCs within the silk hydrogel *in vitro*

### Cell interactions

To observe the cell interactions within the silk hydrogel, we added dropwise 20 μL of silk solutions from three groups (1.0 × 10^5^ cells/mL, 1.0 × 10^6^ cells/mL, 1.0 × 10^7^ cells/mL) into a 96-well plate. The plate was incubated at 37 °C for 10 min for full gelation. Then, 200 μL of DMEM was added into each well and incubated at 37 °C for 24 h before observation with a microscope. For a more comprehensive observation, the encapsulated cells were stained with FITC-phalloidin and DAPI (Invitrogen) and further observed under confocal laser scanning microscopy (CLSM, Leica, Germany).

### Cell proliferation

Cell proliferation activity was evaluated by the MTT cell metabolic assay (Sigma, St. Louis, USA). Silk hydrogels containing different densities of BMSCs were incubated in 96-well plates, as described above. After 1, 4, 7 and 10 days of incubation, the MTT solutions were added into each well and co-incubated with the silk gel for 4 h to form formazan. The formazan was then dissolved with dimethyl sulfoxide, and the optical density (OD) was measured at 490 nm.

### Osteogenic potential *in vitro*

To detect the osteogenic differentiation potential of each study group, we performed an ALP activity assay, a calcium quantification experiment and a qPCR assay. All experiments were performed in triplicate.

For the ALP activity assay, different groups of silk hydrogels were incubated in osteogeneic induced medium for 3 and 7 days. After being fixed in paraformaldehyde for 30 min, the hydrogel was stained with an ALP kit (Beyotime, Shanghai, China) to evaluate the osteogenic potential of the encapsulated cells. Hydrogels with no cells were set as the control in this study.

For the calcium deposition assay, hydrogels were first fixed in neutral formalin at day 21 and then treated with 0.6 N HCL and gently shaken to decalcify for 24 h. The cell lysates were collected and transferred into a 96-well plate, and the cell lysates were incubated with a chromogenic reagent and calcium assay buffer from the calcium assay kit (Sigma, St. Louis, USA) for 10 min away from light. The optical density of the mixed solutions was then measured at 575 nm. A standard curve was set up to define the calcium concentration of each experiment group.

For the real-time quantitative polymerase chain-reaction (qPCR) assay, the total RNA of the cells in silk gel was extracted with Trizol reagent (TaKaRa, Shiga, Japan) after 7 days of incubation and reverse transcribed into cDNA with a PrimeScript 1^st^ strand cDNA synthesis kit (TaKaRa, Shiga, Japan). The qPCR results were measured using a real-time qPCR system (Bio-Rad, Hercules, CA) to evaluate the expression of ALP and OCN genes, with the housekeeping gene GAPDH set for normalization. The final result was calculated using the comparative delta C_t_ method. The primers used in this study were commercially synthesized (Sangon Biotech, Shanghai, China), and the sequences are listed in Table 1.

**Table 1:** Primers for real-time and reverse transcriptase polymerase chain reaction.

Gene	Prime sequence	Product size (bp)	Accession number
*GAPDH*	F:GGCAAGTTCAACGGCACAGT	76	NM_017008.3
	R:GCCAGTAGACTCCACGACAT		
*OCN*	F:GCCCTGACTGCATTCTGCCTCT	103	NM_013414.1
	R:TCACCACCTTACTGCCCTCCTG		
*ALP*	F:GTCCCACAAGAGCCCACAAT	172	NM_013059.1
	R:CAACGGCAGAGCCAGGAAT		

OCN, osteocalcin; ALP, alkaline phosphatase; F, Forward; R, Reverse.

### Surgical procedure for the rat calvarial defect model

A rat calvarial defect model was established, as previously described^25^. Briefly, eighteen F344 rats were anesthetized with pentobarbital through an intraperitoneal injection (3.5 mg/100 g). A 5 mm diameter full-thickness calvarial defect was then created on both sides of the rat’s skull. Different groups of silk gel/scaffold complexes were pre-incubated in osteogenic medium for 7 days to achieve local calcium deposition *in vitro* before random placement into the rat calvarial defects.

### Micro-CT

After 8 weeks of implantation, the rats were sacrificed by injecting an overdose of pentobarbital. The specimens were collected and fixed in 10% buffered formaldehyde solution. The specimens were imaged with a desktop Micro-CT system (μCT-80, Scanco Medical, Switzerland) and scanned in high-resolution mode (pixel matrix, 1024 × 1024; voxel size, 20 μm; slice thickness, 20 μm). We used an image analysis software package (Scanco Medical, Switzerland) to reconstruct the 3D images and detect new bone formation. The new bone volume (BV) and trabecular number (Tb.N) were then analyzed, as previously described^25^. The specimens were further stained with Van Gieson’s picro fuchsin for histological observation.

### Histomorphometric observation

After Micro-CT analysis, the specimens were dehydrated in gradient from 75% to absolute ethanol and embedded in polymethylmethacrylate (PMMA). The specimens were cut into 150 μm thick sections with a Leica SP1600 saw microtome (Leica, Germany) and further polished to a final thickness of 40 μm. The sections were stained with Van Gieson’s picro fuchsin and observed under a confocal laser scanning microscope (CLSM, Leica, Germany). The new bone area was calculated using the Image-Pro Plus^TM^ software program.

### Fluorescence cell tracing experiment

Another 3 rats were included to determine whether the encapsulated osteogenic cells within the silk hydrogel participated in the bone formation process. The cells were labeled with CellTracker^TM^ CM-Dil (Invitrogen, Carlsbad, CA, USA) and then encapsulated into the silk gel/scaffold complex at a density of 1.0 × 10^7^ cells/mL. After incubation in osteogenic medium for 7 days, the complexes were implanted into rat calvarial bone defects. Six weeks after implantation, 20 mg/kg Calcein (Sigma, St. Louis, USA) was intraperitoneally injected into rats to detect new bone formation. At 8 weeks, all rats were euthanized with overdose pentobarbital, and the specimens were harvested. After dehydration, the specimens were embedded in PMMA and then cut and polished into 40 μm thick sections. The sections were further stained with DAPI and observed using a fluorescence stereomicroscope (Leica, Wetzlar, Germany).

### Statistical analysis

The data are all presented as the mean ± standard deviation. ANOVA and SNK post hoc based on the normal distribution and equal variance assumption test were used to test for statistically significant differences (*p* < 0.05; *p* < 0.01) between the different groups in all studies. Statistical analyses were calculated with the SAS 8.2 statistical software package (Cary, USA).

## Electronic supplementary material

Supplementary Information

## References

[1] Damien CJ, Parsons JR (1991). Bone graft and bone graft substitutes: a review of current technology and applications. Journal of applied biomaterials: an official journal of the Society for Biomaterials.

[2] Coombes AG, Meikle MC (1994). Resorbable synthetic polymers as replacements for bone graft. Clinical materials.

[3] Oikarinen, J. & Korhonen, L. K. The bone inductive capacity of various bone transplanting materials used for treatment of experimental bone defects. *Clinical orthopaedics and related research*, 208–215 (1979). 383339

[4] Lane JM, Tomin E, Bostrom MP (1999). Biosynthetic bone grafting. Clinical orthopaedics and related research.

[5] van Gaalen SM (2004). Bone tissue engineering for spine fusion: an experimental study on ectopic and orthotopic implants in rats. Tissue engineering.

[6] Bianco P, Robey PG (2001). Stem cells in tissue engineering. Nature.

[7] Burg KJ, Porter S, Kellam JF (2000). Biomaterial developments for bone tissue engineering. Biomaterials.

[8] Hwang SJ, Cho TH, Kim IS (2014). In vivo gene activity of human mesenchymal stem cells after scaffold-mediated local transplantation. Tissue engineering. Part A.

[9] Chen W (2013). Umbilical cord and bone marrow mesenchymal stem cell seeding on macroporous calcium phosphate for bone regeneration in rat cranial defects. Biomaterials.

[10] Shamsul BS, Tan KK, Chen HC, Aminuddin BS, Ruszymah BH (2014). Posterolateral spinal fusion with ostegenesis induced BMSC seeded TCP/HA in a sheep model. Tissue & cell.

[11] Zimmermann CE (2011). Survival of transplanted rat bone marrow-derived osteogenic stem cells in vivo. Tissue engineering. Part A.

[12] Xu J (2016). Human fetal mesenchymal stem cell secretome enhances bone consolidation in distraction osteogenesis. Stem cell research & therapy.

[13] Kotobuki N (2005). Viability and osteogenic potential of cryopreserved human bone marrow-derived mesenchymal cells. Tissue engineering.

[14] Kotobuki N (2008). In vivo survival and osteogenic differentiation of allogeneic rat bone marrow mesenchymal stem cells (MSCs). Cell transplantation.

[15] Asatrian G, Pham D, Hardy WR, James AW, Peault B (2015). Stem cell technology for bone regeneration: current status and potential applications. Stem cells and cloning: advances and applications.

[16] Meijer GJ, de Bruijn JD, Koole R, van Blitterswijk CA (2007). Cell-based bone tissue engineering. PLoS medicine.

[17] Saeed H (2016). Mesenchymal stem cells (MSCs) as skeletal therapeutics - an update. Journal of biomedical science.

[18] Ohnishi H, Oda Y, Ohgushi H (2010). Stem cell technology using bioceramics: hard tissue regeneration towards clinical application. Science and technology of advanced materials.

[19] Altman GH (2003). Silk-based biomaterials. Biomaterials.

[20] Li M, Ogiso M, Minoura N (2003). Enzymatic degradation behavior of porous silk fibroin sheets. Biomaterials.

[21] Wang Y (2008). In vivo degradation of three-dimensional silk fibroin scaffolds. Biomaterials.

[22] Teimouri A, Azadi M, Emadi R, Lari J, Chermahini AN (2015). Preparation, characterization, degradation and biocompatibility of different silk fibroin based composite scaffolds prepared by freeze-drying method for tissue engineering application. Polymer Degradation and Stability.

[23] Jin HJ, Kaplan DL (2003). Mechanism of silk processing in insects and spiders. Nature.

[24] Omenetto FG, Kaplan DL (2010). New opportunities for an ancient material. Science.

[25] Zhang W (2011). The use of injectable sonication-induced silk hydrogel for VEGF(165) and BMP-2 delivery for elevation of the maxillary sinus floor. Biomaterials.

[26] Zhang W (2014). Porous silk scaffolds for delivery of growth factors and stem cells to enhance bone regeneration. PloS one.

[27] Wang X, Kluge JA, Leisk GG, Kaplan DL (2008). Sonication-induced gelation of silk fibroin for cell encapsulation. Biomaterials.

[28] Yodmuang S (2015). Silk microfiber-reinforced silk hydrogel composites for functional cartilage tissue repair. Acta biomaterialia.

[29] Cushing MC, Anseth KS (2007). Materials science. Hydrogel cell cultures. Science.

[30] Drury JL, Mooney DJ (2003). Hydrogels for tissue engineering: scaffold design variables and applications. Biomaterials.

[31] Johari B (2016). Repair of rat critical size calvarial defect using osteoblast-like and umbilical vein endothelial cells seeded in gelatin/hydroxyapatite scaffolds. Journal of biomedical materials research. Part A.

[32] Fan D (2016). The use of SHP-2 gene transduced bone marrow mesenchymal stem cells to promote osteogenic differentiation and bone defect repair in rat. Journal of biomedical materials research. Part A.

[33] Yassin MA (2017). A Copolymer Scaffold Functionalized with Nanodiamond Particles Enhances Osteogenic Metabolic Activity and Bone Regeneration. Macromolecular bioscience.

[34] Johari B (2016). Osteoblast-seeded bioglass/gelatin nanocomposite: a promising bone substitute in critical-size calvarial defect repair in rat. The International journal of artificial organs.

[35] Minoura N (1995). Attachment and growth of fibroblast cells on silk fibroin. Biochemical and biophysical research communications.

[36] Minoura N, Aiba S, Gotoh Y, Tsukada M, Imai Y (1995). Attachment and growth of cultured fibroblast cells on silk protein matrices. Journal of biomedical materials research.

[37] Gotoh Y, Tsukada M, Minoura N (1998). Effect of the chemical modification of the arginyl residue in Bombyx mori silk fibroin on the attachment and growth of fibroblast cells. Journal of biomedical materials research.

[38] Inouye K, Kurokawa M, Nishikawa S, Tsukada M (1998). Use of Bombyx mori silk fibroin as a substratum for cultivation of animal cells. Journal of biochemical and biophysical methods.

[39] Bellas E (2015). Injectable silk foams for soft tissue regeneration. Advanced healthcare materials.

[40] Wang X, Song W, Kawazoe N, Chen G (2013). The osteogenic differentiation of mesenchymal stem cells by controlled cell-cell interaction on micropatterned surfaces. Journal of biomedical materials research. Part A.

[41] Parekkadan B (2008). Cell-cell interaction modulates neuroectodermal specification of embryonic stem cells. Neuroscience letters.

[42] Charest JL, Jennings JM, King WP, Kowalczyk AP, Garcia AJ (2009). Cadherin-mediated cell-cell contact regulates keratinocyte differentiation. The Journal of investigative dermatology.

[43] Purpura KA, Aubin JE, Zandstra PW (2004). Sustained in vitro expansion of bone progenitors is cell density dependent. Stem cells.

[44] McCulloch CA, Strugurescu M, Hughes F, Melcher AH, Aubin JE (1991). Osteogenic progenitor cells in rat bone marrow stromal populations exhibit self-renewal in culture. Blood.

[45] Kneser U (2006). Evaluation of processed bovine cancellous bone matrix seeded with syngenic osteoblasts in a critical size calvarial defect rat model. Journal of cellular and molecular medicine.

[46] Rao RR, Stegemann JP (2013). Cell-based approaches to the engineering of vascularized bone tissue. Cytotherapy.

[47] Lokmic Z, Mitchell GM (2008). Engineering the microcirculation. Tissue engineering. Part B, Reviews.

[48] Rouwkema J, Rivron NC, van Blitterswijk CA (2008). Vascularization in tissue engineering. Trends in biotechnology.

[49] Zhu C (2011). LvBMP-2 gene-modified BMSCs combined with calcium phosphate cement scaffolds for the repair of calvarial defects in rats. Journal of materials science. Materials in medicine.

[50] Nazarov R, Jin HJ, Kaplan DL (2004). Porous 3-D scaffolds from regenerated silk fibroin. Biomacromolecules.

[51] Kim UJ, Park J, Kim HJ, Wada M, Kaplan DL (2005). Three-dimensional aqueous-derived biomaterial scaffolds from silk fibroin. Biomaterials.

