# Supplementary material for: Increased stem cells delivered using a silk gel/scaffold complex for enhanced bone regeneration
Source: Sci Rep. 2017 May 19;7:2175. doi: 10.1038/s41598-017-02053-z (PMC5438390; doi:10.1038/s41598-017-02053-z)
Supplement: Supplementary file 1 — Supplementary Information [file 41598_2017_2053_MOESM1_ESM.docx]

**Massive stem cells delivered by silk gel/scaffold complex to enhance bone regeneration**

Xun Ding ^1, 2, *^, Guangzheng Yang ^3, *^, Wenjie Zhang ^1, 2, *^, Guanglong Li ^1, 2^, Shuxian Lin ^1, 2^, David L. Kaplan^4^, and Xinquan Jiang ^1, 2, **^

^1^ Department of Prosthodontics, Ninth People’s Hospital affiliated to Shanghai Jiao Tong University, School of Medicine, 639 Zhizaoju Road, Shanghai 200011, China.

^2^ Oral Bioengineering and regenerative medicine Lab, Shanghai Research Institute of Stomatology, Ninth People's Hospital Affiliated to Shanghai Jiao Tong University, School of Medicine, Shanghai Key Laboratory of Stomatology, 639 Zhizaoju Road, Shanghai 200011, China.

^3^ Department of Oral and Maxillofacial Surgery, Ninth People’s Hospital affiliated to Shanghai Jiao Tong University, School of Medicine, 639 Zhizaoju Road, Shanghai 200011, China.

^4^ Department of Biomedical Engineering, School of Engineering, Tufts University, 4 Colby St,Medford, MA 02155, USA.

^*^ These authors contributed equally to this study and share first authorship

^**^ Corresponding author

Prof. Xinquan Jiang,

Ninth People's Hospital, Shanghai Jiao Tong University, School of Medicine, 639 Zhizaoju Road, Shanghai, 200011, China.

Tel.: +86 21 63135412

Fax: +86 21 63136856

E-mail: [xinquanj@aliyun.com](mailto:xinquanj@yahoo.cn)


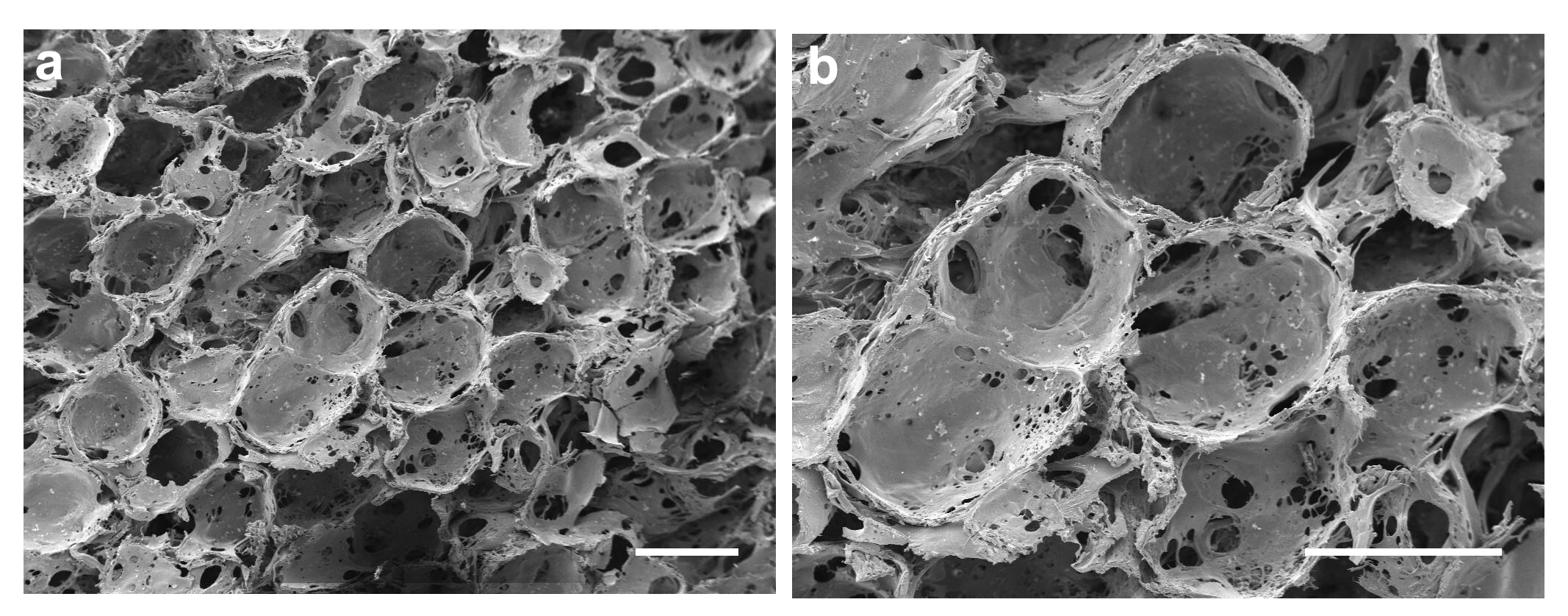


Supplementary Figure. S1 SEM. SEM images of the silk scaffold. Scale bar = 400 μm.

(a) The low magnification SEM image shows the porous silk scaffold with pores size about 350-420 μm. (b) The high magnification image shows that the scaffold is interconnected.

Supplementary Figure. S2 FT-IR. FT-IR spectrum of the silk scaffold.

The infrared (IR) spectral region from 1700 cm^-1^ to 1500 cm^-1^ is widely used to analyze the different secondary structures of silk fibroin, which often includes the absorption by the amide I (1700-1600 cm^-1^) and the amide II (1600-1500 cm^-1^). Our FT-IR spectrum has shown peaks at 1621 cm^-1^ (amide I) and 1514 cm^-1^ (amide II), which are characteristic of silk II secondary structure.
